# Mild metabolic acidosis impairs the β-adrenergic response in isolated human failing myocardium

**DOI:** 10.1186/cc11468

**Published:** 2012-08-13

**Authors:** Hanna Schotola, Karl Toischer, Aron F Popov, André Renner, Jan D Schmitto, Jan Gummert, Michael Quintel, Martin Bauer, Lars S Maier, Samuel Sossalla

**Affiliations:** 1 Department of Anesthesiology, Emergency and Intensive Care Medicine, University Hospital Goettingen, Robert-Koch-Straße 40, Goettingen, 37075, Germany; 2 Division of Cardiology and Pneumology, University Hospital Goettingen, Robert-Koch-Straße 40, Goettingen, 37075, Germany; 3 Department of Thoracic and Cardiovascular Surgery, University Hospital Goettingen, Robert-Koch-Straße 40, Göttingen, 37075, Germany; 4 Department of Cardiothoracic Transplantation and Mechanical Support, Royal Brompton and Harefield Hospital, Hill End Road, London, UB9 6JH, UK; 5 Department of Thoracic and Cardiovascular Surgery, Heart and Diabetes Center, North Rhine Westphalia, Bad Oeynhausen, Georgstraße 11, Bad Oeynhausen, 32545, Germany; 6 Department of Cardiac, Thoracic, Transplantation and Vascular Surgery, Hannover Medical School, Carl-Neuberg-Straße 1, Hannover, 30625, Germany

## Abstract

**Introduction:**

Pronounced extracellular acidosis reduces both cardiac contractility and the β-adrenergic response. In the past, this was shown in some studies using animal models. However, few data exist regarding how the human end-stage failing myocardium, in which compensatory mechanisms are exhausted, reacts to acute mild metabolic acidosis. The aim of this study was to investigate the effect of mild metabolic acidosis on contractility and the β-adrenergic response of isolated trabeculae from human end-stage failing hearts.

**Methods:**

Intact isometrically twitching trabeculae isolated from patients with end-stage heart failure were exposed to mild metabolic acidosis (pH 7.20). Trabeculae were stimulated at increasing frequencies and finally exposed to increasing concentrations of isoproterenol (0 to 1 × 10^-6 ^*M*).

**Results:**

A mild metabolic acidosis caused a depression in twitch-force amplitude of 26% (12.1 ± 1.9 to 9.0 ± 1.5 mN/mm^2^; *n *= 12; *P *< 0.01) as compared with pH 7.40. Force-frequency relation measurements yielded no further significant differences of twitch force. At the maximal isoproterenol concentration, the force amplitude was comparable in each of the two groups (pH 7.40 versus pH 7.20). However, the half-maximal effective concentration (EC_50_) was significantly increased in the acidosis group, with an EC_50 _of 5.834 × 10^-8 ^*M *(confidence interval (CI), 3.48 × 10^-8 ^to 9.779 × 10^-8^; *n *= 9), compared with the control group, which had an EC_50 _of 1.056 × 10^-8 ^*M *(CI, 2.626 × 10^-9 ^to 4.243 × 10^-8^; *n *= 10; *P *< 0.05), indicating an impaired β-adrenergic force response.

**Conclusions:**

Our data show that mild metabolic acidosis reduces cardiac contractility and significantly impairs the β-adrenergic force response in human failing myocardium. Thus, our results could contribute to the still-controversial discussion about the therapy regimen of acidosis in patients with critical heart failure.

## Introduction

For more than 100 years, pronounced extracellular acidosis has been known to depress contractility in the healthy myocardium [1-3]. The mechanisms underlying this negative inotropic effect, however, are complicated by the fact that changing the pH can modify many of the cellular systems involved in the excitation-contraction (EC) coupling pathway, including both the delivery of Ca^2+ ^to the myofilaments and the Ca^2+ ^sensitivity of the myofilaments [4-10]. Several Ca^2+ ^transport systems are depressed at a low pH, including the SR Ca^2+^-ATPase (SERCA), the ryanodine receptor (RyR), and the Na^+^/Ca^2+ ^exchanger (NCX) [6]. Therefore, acidosis has been shown to decrease the cellular Ca^2+ ^transient amplitude [1]. Kohlhardt and co-workers [11] showed a significantly decreased cardiac output induced by acidosis (pH 7.00) by almost 25% in nonfailing cat hearts. An additional number of studies verified the decrease in contractility in other species [12-18]. Studies have also demonstrated that the beta-adrenergic response is reduced by acidosis, although the number of studies regarding this subject is limited [19-23].

Most of the studies investigating the effects of acidosis on cardiac contractility were performed with rather low pH values (for example, pH ≥7.00), which might be out of the range that commonly and frequently occurs in clinical practice, such as in peri- and postcardiac surgery. Moreover, to our knowledge, little is known regarding the contractile behavior *in vitro *of the human myocardium under mild metabolic acidotic conditions. We recently showed that a mild and thus clinically relevant metabolic acidosis (pH 7.20) had no significant influence on the cardiac contractility and isoproterenol response in isolated trabeculae of the nonfailing ovine myocardium [24].

However, heart-failure patients are prone to develop metabolic acidosis (for example, because of prolonged extracorporeal circulation during cardiac surgery). In on-pump surgery, pH changes are often observed, for example, as a result of volume shifts and the systemic inflammatory response syndrome (SIRS) [25-27]. Patients with severe heart failure are first treated conservatively and, at some stage, with transplantation or left ventricular assist device [28]. These patients represent a special group that must be treated with care and safety. After a long-standing illness, the compensatory mechanisms of these patients are often fully exhausted, and hence, these patients may react differently and/or earlier to pathophysiologic conditions. Moreover, heart-failure patients often require acute catecholaminergic therapy both during and after cardiac surgery. However, the beta-adrenergic response under mild metabolic acidosis has, to our knowledge, never been investigated in isolated human failing myocardium. Therefore, the first aim of our study was to explore how the contractility of the human failing myocardium reacts to mild metabolic pH changes. Moreover, and most important, the second aim was to investigate the clinical relevance of the beta-adrenergic response under mild metabolic acidosis, possibly to contribute basic knowledge to the controversy surrounding the therapy regimen of mild metabolic acidosis in critical heart-failure patients.

## Materials and methods

### Human failing myocardium

Eight end-stage human failing hearts were obtained from patients undergoing heart transplantation. Inclusion criteria were diagnosed terminal heart failure, listing for transplantation (Eurotransplant criteria) due to dilated or ischemic cardiomyopathy. Other cardiomyopathies were excluded. The ejection fraction should have been less than or equal to 30%. Patients were included when they were older than 18 years. Paired experiments that always consisted of two trabeculae isolated from the same human heart were performed by using 24 isolated ventricular trabeculae. All patients received pharmacologic treatment consisting of angiotensin-converting enzyme inhibitors/AT_1_-receptor antagonists, β-adrenoceptor antagonists, diuretics, digitalis, and/or antiarrhythmic agents. Patients' clinical characteristics and drug regimens are listed in Table 1. During surgery, all patients received anesthetics and antibiotic prophylaxis. The ethical committee of the University Hospital of Goettingen approved these experimental procedures with human tissue. Furthermore, informed consent was given by all patients for using their heart for research purposes.

**Table 1:** Heart-failure patients' clinical characteristics and previous drug regimens

	Sex	Age (years)	EF (%)	PCW (mm Hg)	CI (L/min/m^2^)	ACE-I	AT_1_	β-B	DIU	DIG	AMIOD
ICM	m	51	20-25	n.a.	n.a.	+	-	-	+	-	-
DCM	m	41	25	17	2.14	+	-	+	+	-	-
DCM	m	58	5-10	26	2.09	-	+	+	+	+	-
ICM	m	52	15	n.a.	n.a.	+	-	+	-	-	-
ICM	m	63	20	20	2.24	+	-	+	+	-	+
DCM	f	57	15	n.a.	2.21	-	+	+	+	-	-
DCM	m	32	20	5	2.64	+	-	+	+	+	-
ICM	f	59	23	11	2.21	+	-	-	+	-	+

ACE-I, angiotensin-converting enzyme inhibitor; AMIOD, amiodarone; AT_1_, AT_1_-receptor antagonist; β-B, β-blocker; CI, cardiac index; DCM, dilated cardiomyopathy; DIG, digitalis; DIU, diuretic; EF, ejection fraction; ICM, ischemic cardiomyopathy; PCW, pulmonary capillary wedge pressure.

### Muscle strip preparation

After explantation, hearts were stored and transported in an iced-cooled cardioprotective solution (m*M*) consisting of Na^+ ^152, K^+ ^3.6, Cl^− ^135, HCO^3− ^25, Mg^2+ ^0.6, H_3_PO^4− ^1.3, SO_4_^2− ^0.6, Ca^2+ ^2.5, glucose 11.2, and 2,3-butanedione monoxime (BDM) 10. The tissue was oxygenated with 95% O_2 _and 5% CO_2_. Thin ventricular trabeculae were prepared as described previously [29]. Afterward, the trabeculae were isolated from the right ventricle because of the high endocardial fibrosis that is present in the left ventricle of human end-stage failing hearts. In a dissection chamber containing a BDM-solution, ventricular trabeculae were prepared from the same area of the hearts by using stereoscopic microscopy [30,31].

### Isometric force recordings

For isometric force recordings, the trabeculae were mounted in a superfusion organ chamber between a force transducer (Scientific Instruments, Heidelberg, Germany) and a hook connected to a micromanipulator for length adjustment. As previously described, the trabeculae were superfused with a HEPES (4-(2-hydroxyethyl)-1-piperazineethanesulfonic acid) buffering solution, pH 7.40 (m*M*: NaCl 116, KCl 5, NaH_2_PO_4 _2, MgCl_2 _1.2, Na_2_SO_4 _1.2, NaHCO_3 _20, glucose 10, CaCl_2 _initially at 0.25 and after with a stepwise increase of 2.0), at a temperature of 37°C and oxygenated with 100% O_2 _[24]. Contractions were caused by electrical field stimulation (baseline, 1 Hz; 5 to 7 mV; STIM1, Scientific Instruments). At 5-minute intervals, 0.25 m*M *Ca^2+ ^was added until the final concentration of 2.0 m*M *was reached. After 30 minutes of equilibration time with an initial prediastolic tension of 0.1 mN/mm^2^, the trabeculae were gradually stretched until reaching the maximum steady-state twitch force to improve comparability. When steady-state twitch force was achieved, the superfusion was completely changed with a HEPES solution with a pH of either 7.40 (control group) or 7.20 (mild acidosis group) (37°C, CaCl_2 _2.0 m*M*, oxygenated with 100% O_2_). After reaching a steady-state force again, force-frequency relation (FFR) measurements were obtained by using stimulation frequencies of 1, 2, and 3 Hz. To measure the β-adrenergic response, the trabeculae were exposed to increasing concentrations of (±)-isoproterenol (Sigma Aldrich, Munich, Germany) up to 1 × 10^-6 ^*M *at a stimulation frequency of 1 Hz. A custom-made software on the LabVIEW platform (National Instruments Corporation, Austin, TX, USA) was used to record and analyze the force of the isometric contractions.

### Statistical analysis

Detected force values were normalized to the cross-sectional area of each trabecula (width × thickness ×π/4) and are presented as either systolic force or diastolic tension in mN/mm^2^. Data are presented as mean ± standard error of the mean (SEM) or confidence intervals (CIs) for EC_50 _values. The Student paired *t *test or a two-way repeated-measures ANOVA was performed to test for statistically significant differences. A value of *P *< 0.05 was considered to be statistically significant. Statistics and curve fits were obtained with GraphPad Prism 5 software (GraphPad Software, Inc., La Jolla, CA, USA).

## Results

### Baseline conditions

No statistical difference in the cross-sectional dimensions (width × thickness × 4/π) between the two pH groups was detectable (pH 7.40 group, 0.61 ± 0.09 mm^2 ^(*n *= 7), versus pH 7.20 group, 0.61 ± 0.11 mm^2 ^(*n *= 12); *P *= 0.97). Twitch-force amplitude was not statistically different before the pH solution change at 1 Hz in both groups, with 13.9 ± 3.1 mN/mm^2 ^for the pH 7.40 control group and 12.1 ± 1.9 mN/mm^2 ^for the pH 7.20 group (*n *= 7 versus *n *= 12, *P *= 0.65). For further analyses, force was normalized to baseline values at 1 Hz to better distinguish the effects of acidosis on myocardial contractility.

### Immediate effects of mild acidosis on basal contractility

**Figure 1:**
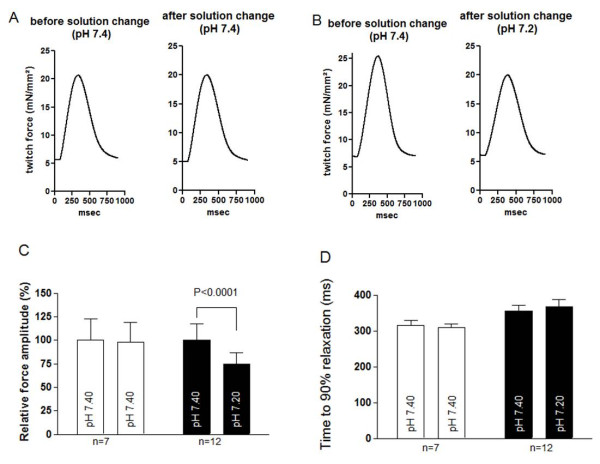
**Immediate changes in pH**. **(A) **Representative single twitches before and after changing extracellular pH. No obvious reduction of developed tension is visible. **(B) **Representative single twitches after an acute pH change show a decrease of absolute developed tension due to mild metabolic acidosis. **(C) **Mean values of relative force amplitude before and after pH change show a significant decrease of force amplitude after changing to the mild acidotic solution. **(D) **Time to 90% relaxation before and after pH solution change yielded no statistical difference.

To measure the immediate changes in contractile behavior with exposure to a mild acidotic pH, we changed the solution to a HEPES solution with a pH of 7.20. Figures 1A and 1B show the original twitch-recordings of representative single twitches before and after the pH changes. In the control group, the solution was also changed to a fresh solution with pH 7.40 to minimize unspecific effects (for example, those due to temperature differences). Our results show that the mild acidotic pH caused a statistically significant decrease in the twitch force amplitude from 12.1 ± 1.9 to 9.0 ± 1.5 mN/mm^2^, which corresponds to a negative inotropic effect of 26% (*n *= 12; *P *< 0.01) (Figure 1C). However, no significant decline was observed in the twitch force within the control group (13.9 ± 3.1 to 13.6 ± 2.9 mN/mm^2^; *n *= 7; *P *= 0.50) (Figure 1C). Relaxation time (Figure 1D) and relaxation velocity (data not shown) did not statistically differ between pH 7.40 and pH 7.20.

### Force-frequency relation

**Figure 2:**
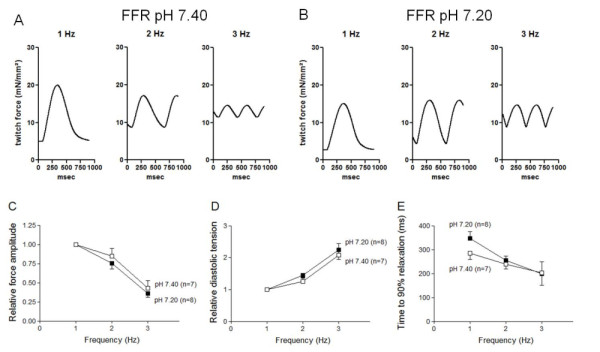
**Force-frequency relation. (A, B) **Representative single twitches show a decrease of force amplitude and an increase of diastolic tension in both groups. **(C) **Relative force amplitudes at frequencies between 1 Hz and 3 Hz show a negatively shaped force-frequency relation. **(D) **Diastolic tension normalized to the values recorded at the lowest frequency revealed no differences between the two groups. **(E) **Mean values of time to 90% of relaxation do not differ significantly between the two pH groups.

The force-frequency relation (FFR) was measured at frequencies between 1 and 3 Hz. Original registrations of isometrically twitching trabeculae in the presence of a physiologic pH of 7.40 compared with a mild acidotic pH of 7.20 are presented in Figure 2A and 2B. Both groups showed a negatively shaped FFR, whereas stimulation frequencies increased, as is typical for the failing human myocardium (Figure 2C) [32]. Force amplitudes decreased by 15% ± 10% for pH 7.40 and by 25% ± 8% for pH 7.20 at 2 Hz and by 57% ± 10% and by 64% ± 8% at 3 Hz, compared with 1 Hz (*n *= 7 versus *n *= 8; *P *= 0.30). For diastolic tension, the curves showed a marked increase between 1 and 3 Hz (Figure 2D), representing the diastolic dysfunction in the failing myocardium. Moreover, the diastolic tension increased by 26% ± 11% for pH 7.40 and by 39% ± 6% for pH 7.20 at 2 Hz, and by 109% ± 35% for pH 7.40 and 105% ± 25% for pH 7.20 at 3 Hz (*n *= 7 versus *n *= 8; *P *= 0.48). During increasing frequencies, the trabeculae had faster relaxation times. However, relaxation parameters (such as time to 90% relaxation, Figure 2E) did not significantly differ between the two pH groups (pH 7.40, *n *= 7, versus pH 7.20, *n *= 8; *P *= 0.40).

### β-Adrenergic response

#### Maximal twitch force

**Figure 3:**
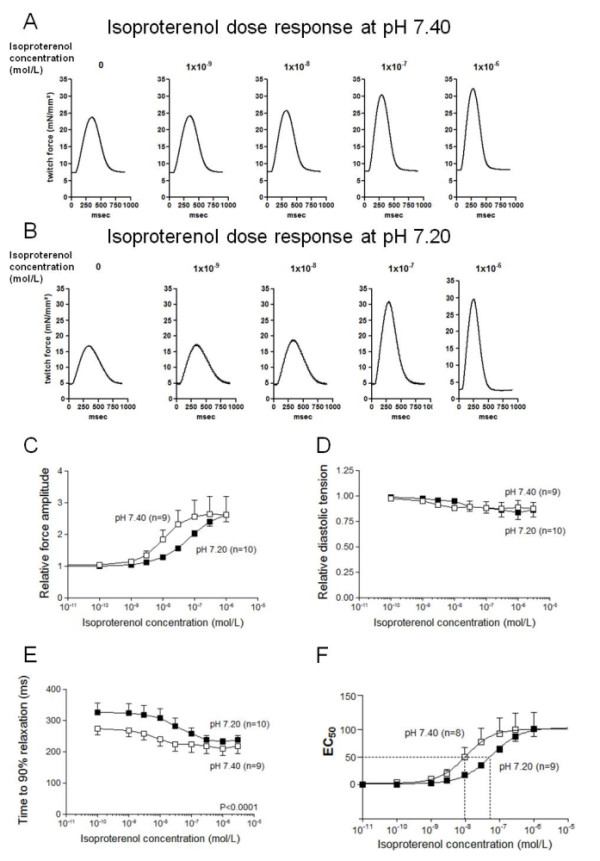
**β-Adrenergic response**. **(A, B) **Representative single twitches show an increase of force amplitude with increasing isoproterenol concentrations in both groups, but faster in the pH 7.4 group. **(C) **Relative force amplitudes normalized to baseline force showed a sigmoid shape with increasing isoproterenol. **(D) **Diastolic tension normalized to baseline revealed no differences between the groups: the tension decreased minimally with increasing isoproterenol. **(E) **Time to 90% of relaxation was statistically significantly slower in the mild acidotic group than in the control group with pH 7.40. **(F) **Half-maximal effective concentration (EC_50_) was statistically significantly different between the pH groups.

To verify the effect of mild metabolic acidosis on the β-adrenergic response in the isolated human failing myocardium, the trabeculae were exposed to increasing concentrations of the β-adrenergic agonist isoproterenol. In Figure 3A and 3B, representative original registrations are depicted in the presence of increasing concentrations of isoproterenol up to 1 × 10^-6 ^*M*. In both groups, the force amplitude increased with the increasing concentrations of isoproterenol until it reached a plateau (Figure 3C). Twitch-force amplitudes that were normalized to baseline increased by 160% ± 50% for pH 7.40 and by 161% ± 21% for pH 7.20 at the maximal isoproterenol concentration of 1 × 10^-6 ^*M *(*n *= 9 versus *n *= 10; *P *= 0.99; Figure 3C). Thus, the maximal twitch force in response to isoproterenol was not significantly different between the two pH groups (Figure 3C). Accordingly, the diastolic tension did not differ between the groups (Figure 3D). In the control group, a decrease of 13% ± 7% occurred at the maximal β-adrenergic stimulation, and, in the mild metabolic pH group, a decrease of 16% ± 7% was seen, which reflects a physiologic run-down due to the myofilament relaxation after maximal stretching (pH 7.40; *n *= 9 versus pH 7.20; *n *= 10; *P *= 0.69; Figure 3D). Analyses of the relaxation kinetics (time to 90% relaxation) yielded a statistically slower relaxation in the mild acidotic group than in the control group, with pH 7.40 (at baseline: 329 ± 29 ms (pH 7.20) versus 273 ± 18 ms (pH 7.40), at maximal isoproterenol concentration: 237 ± 12 ms (pH 7.20) versus 218 ± 22 ms (pH 7.40); *n *= 9 versus *n *= 11; *P *< 0.1; Figure 3E).

#### Determination of the EC_50 _

The maximum of the β-adrenergic force response was not statistically different between a normal and a mild acidotic pH, as described earlier. However, from a clinical point of view, it is important to obtain more information regarding the β-adrenergic force response within the ascending part of the dose-response curve. Therefore, we also determined the half-maximal effective concentration (EC_50_). A significant rightward shift of the pH 7.20 curve was found (pH 7.40, *n *= 8 versus pH 7.20, *n *= 8; *P *< 0.05). The EC_50 _was significantly increased to 5.834 × 10^-8 ^*M *(CI, 3.48 × 10^-8 ^to 9.779 × 10^-8 ^*M*) compared with the control group, with 1.056 × 10^-8 ^*M *(CI, 2.626 × 10^-9 ^to 4.243 × 10^-8 ^*M, n *= 10 versus *n *= 9, *P *< 0.05, Figure 3F). Thus, the β-adrenergic response of the isometrically contracting human failing myocardium is significantly depressed under mild acidosis.

## Discussion

The aim of this study was to investigate the extent to which mild metabolic acidosis with a pH of 7.20 influences cardiac contractility and the β-adrenergic response in human end-stage failing myocardium.

Our results show that acute mild acidosis impairs contractility and the β-adrenergic response in trabeculae isolated from human end-stage failing hearts. Changing the superfusion from a physiologic pH to mild acidosis (pH 7.20) directly caused a significant depression in the twitch-force amplitude. However, a mild acidotic pH had no additional effect on the contractile behavior during increasing frequencies (1 to 3 Hz). Although the force amplitude at maximal concentrations of isoproterenol was not altered by mild acidosis, a pH of 7.20 caused a rightward shift of the isoproterenol dose response curve, leading to a significantly increased EC_50_.

### Immediate pH changes

Over the last decades, the influence of metabolic acidosis on cardiac contractility was intensively investigated in different animal models [1,2,4,5,9,11,12,33]. In the presence of an acidotic pH, a serious species-independent decrease in contractility was found [11]. Therefore, acidosis influences EC coupling and the Ca^2+^-myofilament response [4,5,7-10]. Studies have shown that acidosis inhibits almost every step of the cellular EC coupling: Kentish and Xiang [15,17,18,34] found a reduced Ca^2+ ^release from the SR via RyR and a direct inhibition of the SERCA by acidosis that slows Ca^2+ ^uptake into the SR and found that the direct inhibition of NCX by a pathologically low pH increases the amount of intracellular Ca^2+^. This disturbance in the Ca^2+^-myofilament response acidosis leads to a decrease in the apparent sensitivity of the regulatory sites of troponin C to Ca^2+ ^[2,13].

In the human failing myocardium, cardiac contractility is reduced, and compensatory mechanisms are exhausted. Cardiomyocytes of patients with end-stage heart failure show a reduced SERCA activity and a downregulation of the SERCA protein, whereas NCX activity was shown to be upregulated [32,35-37]. These systems compete with each other, leading to a cellular Ca^2+ ^loss that is further aggravated by the leakiness of the RyR in heart failure [29]. Therefore, the failing myocardium is much more susceptible to negative inotropic effects because of a blunted contractile reserve subsequent to decreased sympathetic sensitivity or a negative force-frequency relation. In addition, the dependence of H^+ ^elimination from H^+^/K^+^-ATPase may be increased in heart failure because of the impaired function of the Na^+^/H^+ ^exchange subsequent to increased [Na^+^]_i _[38].

Recently, we showed that an acute mild metabolic pH shift to 7.20 did not influence cardiac contractility in the isolated trabeculae of nonfailing ovine hearts [24]. The results of the current study with end-stage human failing myocardium show a significant decrease in force amplitude under comparable conditions. Because compensatory mechanisms are exhausted in heart failure after long-lasting intracellular remodeling, it seems that acidotic effects cannot be adequately buffered.

### Force-frequency relation

In our experiments, a negative force-frequency relation and an abnormal increase in diastolic tension with increasing stimulation frequencies was observed. Failing human myocardium is well known to show this negative force-frequency relation [39,40]. Hasenfuss *et al*. [39] showed that the altered FFR results from a disturbed excitation-contraction coupling as a result of reduced SERCA activity [39]. At higher stimulation frequencies, this reduced activity leads to a decreased calcium cycling [40]. Thus, it could be suggested that mild metabolic acidosis might induce further negative inotropic effects during increasing stimulation frequencies. Conversely, in our experiments, acidosis was already present at 1-Hz basal pacing frequency, causing a negative inotropic effect. Thus it could also be suggested that there might not be additional effects on top of this. Indeed, we only observed basal negative inotropic effects at 1-Hz stimulation frequency, which was not further aggravated by increasing stimulation frequencies.

We found no data in the literature in which the direct effect of acidosis on the FFR was investigated, especially not in the human heart failing myocardium. Morii *et al*. [41] showed in their work that in intact rat ventricular myocardium at increasing frequencies (3 to 5 Hz), the intracellular pH decreased (pH <7.10). This pH decrease generates a reduced Ca^2+ ^sensitivity and yields a negative FFR [41]. Our data suggest that, in contrast, a mild metabolic acidosis does not further enhance the negative inotropic effect in FFR in human end-stage failing myocardium.

### β-Adrenergic response

In patients with severe heart failure, a prolonged increase in the activation of the sympathetic nervous system and therefore increased β-adrenergic hormone blood levels leads to a desensitization to adrenergic stimulation [42]. This desensitization reduces the contractile reserves for physical exertion and is also implemented by a selective downregulation of myocardial β_1_-adrenergic receptors in heart failure [43-46].

In our study, we found a delayed response to increasing isoproterenol concentrations under acidotic conditions, whereas the β-adrenergic maximal reached force amplitude did not differ between the two pH groups. Acidosis changes the relative binding affinities of inotropes and vasopressors to adrenergic receptors [42]. A study that investigated the effect of metabolic acidosis regarding the response to different catecholamines showed that catecholamines (norepinephrine, epinephrine, isoproterenol, and phenylephrine) react unequally to mild metabolic acidosis in a large-animal model [47]. They found that in the presence of acidosis, epinephrine was the most affected and that isoproterenol, as a potent, nonselective, synthetic β-adrenergic agonist, was the least affected by mild changes in pH.

The present study is, to our knowledge, the only series of experiments that has investigated the catecholamine response in end-stage failing human myocardium in the presence of a mild metabolic acidosis. We could show this effect by using isoproterenol. However, to get information about the behavior of other catecholamines, an additional series should follow. .

Therefore, this specially modified and extremely fragile myocardium requires greater β-adrenergic stimuli to reach the same force amplitude when the pH is mildly decreased compared with a normal pH.

### Clinical influence and relevance

The prolonged use of positive inotropes in patients with chronic heart failure is well known to increase mortality [48,49]. Hence, the American College of Cardiology/American Heart Association and the European Society of Cardiology recommend in their guidelines for the diagnosis and management of chronic heart failure in the adult that intravenous inotropic agents should not be used routinely for patients with refractory end-stage heart failure but for palliation of symptoms in these patients [50,51]. One should consider that patients with terminal heart failure are a very special and sensitive patient group with a reduced capacity to react to β-adrenergic stimulation.

Particularly critical times for heart-failure patients undergoing on-pump cardiac surgery are those during and after weaning from the cardiopulmonary bypass, because a low cardiac-output syndrome can occur. Because of cardiac ischemia, myocardial dysfunction induced by cardioplegia, reperfusion injury, the presence of nonrepaired preexisting cardiac disease, and the activation of coagulation cascades and inflammation, a pharmacologic support can be necessary [52]. It is not unusual for these patients to develop a systemic inflammatory response syndrome (SIRS) that may lead to sepsis [25-27]. In this context, pH changes are quite common. The sepsis guidelines of different societies do not recommend buffering when pH is higher than or equal to 7.15 to counteract the decrease in cardiac contractility because the application of a pH buffer like bicarbonate can also have negative side effects [53-56]. The guidelines are also restrained to recommend a therapy for a pH lower than 7.15. However, our data show that in the end-stage heart, failing myocardium mild metabolic acidosis (pH 7.20) already depresses contractility and causes a delayed β-adrenergic response that might be of clinical relevance. This special patient group often has an earlier and more pronounced reaction to pathophysiologic conditions, and therefore, patients in this group should be treated individually according their underlying diseases.

### Limitations

This study has several limitations. All hearts were explanted as a result of organ transplantation for end-stage heart failure. Therefore, this patient group is very special, and its incidence in the clinical routine is not common. Normally, cardiac surgery patients have a more moderate form of heart failure in which some compensatory mechanisms still work. Furthermore, we artificially induced acidosis *in vitro *from which we derived direct information about the effects of selective acidosis on myocardial contractility. Therefore, our results should be translated into a clinical situation with cautiousness because acidosis *in vivo *is a part of a complex process of myocardial impairment during shock conditions and is associated with proinflammatory cytokines and NO generation.

The data are preliminary and should be confirmed in an *in vivo *model followed by a large clinical study.

However, to our knowledge, this is the first investigation that shows that an acute mild metabolic acidosis has already had a negative influence on cardiac contractility and the β-adrenergic response in the isolated myocardium of end-stage heart failure patients.

## Conclusions

In the end-stage heart failing myocardium, a preexisting acute mild metabolic acidosis with a pH of 7.20 leads to a decrease in contractility and a delayed β-adrenergic response. Our results may contribute to the controversial discussion about the therapy regimen of acidosis, particularly in this special patient group.

## Key messages

• The present study shows that clinically relevant low metabolic acidosis impairs myocardial contractility in isolated human failing myocardium.

• Low metabolic acidosis delays the β-adrenergic response of human failing myocardium.

• From our point of view, it seems to be important to recognize patients with severe heart failure as a special patient group that reacts very sensitively to pH changes because of already exhausted compensatory mechanisms.

• Our study should trigger clinical trials to investigate possible treatment strategies for patients with heart failure in the critical situation of metabolic acidosis.

## Abbreviations

AT_1_: Angiotensin II receptor: type 1; ATP: adenosine triphosphate; BDM: 2,3-butanedione monoxime; Ca^2+^: calcium; CaCl_2_: calcium chloride; CI: confidence interval; Cl^-^: chloride; CO_2_: carbon dioxide; EC: excitation-contraction; EC_50_: half-maximal effective concentration; FFR: force-frequency relation; H_3_PO_4_^−^: phosphoric acid; HCO_3_^−^: bicarbonate; HEPES: 4-(2-hydroxyethyl)-1-piperazineethanesulfonic acid; K^+^: potassium; KCl: potassium chloride; Mg^2+^: magnesium; MgCl_2_: magnesium chloride; Na^+^: sodium; NaCl: sodium chloride; NaHCO_3_: sodium hydrogen carbonate; NaH_2_PO_4_: monosodium phosphate; Na_2_SO_4_: sodium sulfate; NCX: Na^+^/Ca^2+ ^exchanger; O_2_: oxygen; pH: negative decimal logarithm of the hydrogen ion activity in a solution; RyR: ryanodine receptor; SEM: standard error of the mean; SERCA: SR Ca^2+^-ATPase; SIRS: systemic inflammatory response syndrome; SO_4_^2−^: sulfate; SR: sarcoplasmic reticulum.

## Competing interests

All authors declare that they have no conflict of interest regarding the present study. The authors have full control of primary data and confirm that in case of interest, the requisite data will be provided.

## Authors' contributions

HS performed the experiments, performed the statistical analysis, and wrote the manuscript. KT performed the experiments and coordinated the logistic part of the study. AFP made substantial contributions to heart supply, analysis of data, and helped to draft the manuscript and revision. AR explanted the hearts of terminal heart-failure patients undergoing heart transplantation and helped to draft the manuscript. JDS supplied failing hearts and helped to draft the revision substantially. JG explanted the hearts of terminal heart-failure patients undergoing heart transplantation and helped to draft the manuscript. MQ and MB helped to draft the manuscript and revision. LSM participated in its design and helped to draft the manuscript. SS conceived of the study, participated in its design and coordination, and helped to draft the manuscript. All authors read and approved the final manuscript.

## Authors' information

Dr. Sossalla is funded by the Research Program, Faculty of Medicine, Georg-August-University of Göttingen, Germany. Dr. Maier is funded by the Deutsche Forschungsgemeinschaft (DFG) through the Clinical Research group KFO155 (MA 1982/2-2) and a Heisenberg grant (MA 1982/4-1).
